# A Self-Guided Web-Based App (MyDiaMate) for Enhancing Mental Health in Adults With Type 1 Diabetes: Insights From a Real-World Study in the Netherlands

**DOI:** 10.2196/52923

**Published:** 2024-04-03

**Authors:** Jiska Embaye, Maartje de Wit, Frank Snoek

**Affiliations:** 1 Department of Medical Psychology, Amsterdam Public Health Amsterdam UMC Vrije Universiteit Amsterdam Amsterdam Netherlands

**Keywords:** type 1 diabetes, e-mental health, web based, self-help, real world, naturalistic, uptake, adoption, usage, mental health, distress, emotional well-being, cognitive behavioral therapy, internet-based cognitive behavioral therapy, Europe, Netherlands, Dutch

## Abstract

**Background:**

MyDiaMate is a web-based intervention specifically designed for adults with type 1 diabetes (T1D) that aims to help them improve and maintain their mental health. Prior pilot-testing of MyDiaMate verified its acceptability, feasibility, and usability.

**Objective:**

This study aimed to investigate the real-world uptake and usage of MyDiaMate in the Netherlands.

**Methods:**

Between March 2021 and December 2022, MyDiaMate was made freely available to Dutch adults with T1D. Usage (participation and completion rates of the modules) was tracked using log data. Users could volunteer to participate in the user profile study, which required filling out a set of baseline questionnaires. The usage of study participants was examined separately for participants scoring above and below the cutoffs of the “Problem Areas in Diabetes” (PAID-11) questionnaire (diabetes distress), the “World Health Organization Well-being Index” (WHO-5) questionnaire (emotional well-being), and the fatigue severity subscale of the “Checklist Individual Strength” (CIS) questionnaire (fatigue). Two months after creating an account, study participants received an evaluation questionnaire to provide us with feedback.

**Results:**

In total, 1008 adults created a MyDiaMate account, of whom 343 (34%) participated in the user profile study. The mean age was 43 (SD 14.9; 18-76) years. Most participants were female (n=217, 63.3%) and higher educated (n=198, 57.6%). The majority had been living with T1D for over 5 years (n=241, 73.5%). Of the study participants, 59.1% (n=199) of them reported low emotional well-being (WHO-5 score≤50), 70.9% (n=239) of them reported elevated diabetes distress (PAID-11 score≥18), and 52.4% (n=178) of them reported severe fatigue (CIS score≥35). Participation rates varied between 9.5% (n=19) for social environment to 100% (n=726) for diabetes in balance, which opened by default. Completion rates ranged from 4.3% (n=1) for energy, an extensive cognitive behavioral therapy module, to 68.6% (n=24) for the shorter module on hypos. There were no differences in terms of participation and completion rates of the modules between study participants with a more severe profile, that is, lower emotional well-being, greater diabetes distress, or more fatigue symptoms, and those with a less severe profile. Further, no technical problems were reported, and various suggestions were made by study participants to improve the application, suggesting a need for more personalization.

**Conclusions:**

Data from this naturalistic study demonstrated the potential of MyDiaMate as a self-help tool for adults with T1D, supplementary to ongoing diabetes care, to improve healthy coping with diabetes and mental health. Future research is needed to explore engagement strategies and test the efficacy of MyDiaMate in a randomized controlled trial.

## Introduction

Living with and self-managing type 1 diabetes (T1D) can be psychologically burdensome. Indeed, diabetes-related distress (diabetes distress) [1-3], depression [4], fatigue [5], and disordered eating [6] are frequently experienced by people with T1D. Emotional distress is associated with reduced quality of life and can negatively affect diabetes self-care and subsequent glycemic outcomes [7].

The significance of addressing mental health issues in diabetes care has gained increasing recognition over the years. Accordingly, psychological interventions specifically tailored to reduce psychological distress related to diabetes have been developed and shown to be effective [8,9]. These interventions may be more widely available when provided digitally, especially in settings where there is limited access to professional psychological support [10]. Self-guided digital interventions, that is, without any professional involvement, may help to expand reach at relatively low costs. Such digital self-help programs would be particularly suited for people with mild to moderate symptoms of distress, and with the advantage of providing flexibility and anonymity which could attract users who are normally unable or unwilling to seek help [11-14]. However, uptake and engagement with self-guided applications for mental health may be challenging [15].

Over the past years, numerous digital interventions for people with diabetes have been developed focusing on lifestyle changes and blood glucose control, that do not address coping with the psychological burden of T1D [16]. To fill this gap, we worked with end users and professionals to develop MyDiaMate, a fully self-guided web-based intervention specifically designed for adults with diabetes that aims to help them maintain and improve their mental health. MyDiaMate was pilot-tested, confirming its acceptability, feasibility, and usability [17].

Before evaluating the efficacy of MyDiaMate and subsequently embedding MyDiaMate into routine diabetes care, it can be useful to examine its performance in a naturalistic setting. This can improve our understanding of the potential uptake, user profiles, and user behaviors that can give directions to the further development of effective strategies for engagement and dissemination [12,18]. The main purpose of this study, therefore, was to investigate the uptake and usage of MyDiaMate in the Netherlands for the duration of 21 months. To gain more insight into the characteristics and experiences of the users, we offered the option to participate in a user profile study. This would allow us to explore the associations between user characteristics and user behaviors.

## Methods

### MyDiaMate

MyDiaMate is a web-based, multimodular self-help application, designed to assist adults with diabetes in preserving and improving their mental health. The development process of the app and content has previously been described in detail [17]. MyDiaMate is largely psychoeducational in nature and covers a range of topics known to be sources of diabetes distress—coping with the daily demands of self-managing diabetes; fear of hypo’s and worries about complications; problems around social interactions and communication with others, including medical professionals; and 2 more in-depth modules tapping into “Mood” and “Energy,” both of which are based on guided internet-based (cognitive behavioral therapy) interventions for people with diabetes, depression, and fatigue, respectively [5,19]. “Diabetes in Balance” is presented as the starting module and finishes with the recommendation to proceed with any of the following modules in any preferred order.

**Figure 1:**
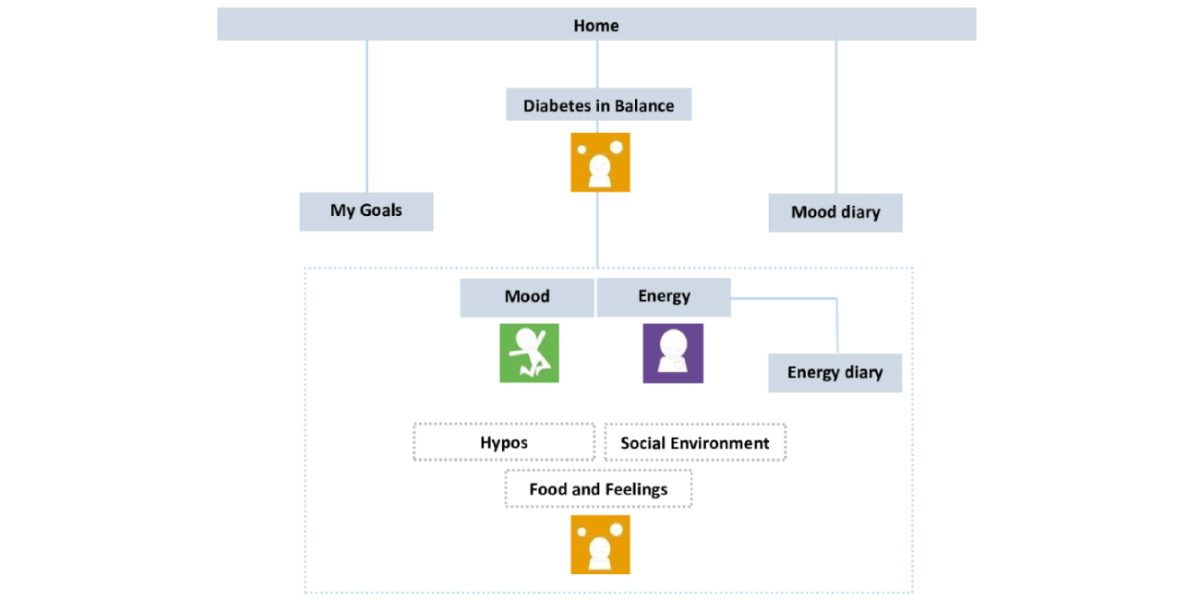
Content and structure of release 1 and release 2 of MyDiaMate. In release 2, the modules hypos and social environment were separated from the diabetes in balance module, and the food and feelings module was added.

Originally intended for individuals with type 1 or type 2 diabetes, MyDiaMate’s content was modified to specifically target T1D, based on user feedback in the pilot study. In December 2021, we launched a second version of the app based on user feedback, where we reduced the density of content in the diabetes in balance module by separating out the sections on “Social Environment” and “Hypos,” and added a “Food and Feelings” module addressing problematic eating in relation to diabetes, and included 9 patient testimonial videos to enhance user engagement across different modules. Figure 1 visually demonstrates the app’s 2 versions.

MyDiaMate is offered on an eHealth platform, using the Minddistrict Content Management System [20]. It can be used on a laptop, tablet, or a mobile phone (iOS and Android) as preferred. The program offers different features known to promote active use, such as goal setting, exercises, tips, quotes, milestones, a mood diary, and an energy diary including notifications, and links to resources. “My Goals” is a tool that can be used parallel to the other modules, to help formulate a personal goal for the duration of the program. At the end of the different modules, users are offered self-reflection questions, that prompt an answer to help the user decide what next step to take—to continue working in a specific module, to promote further progress, or engage in other modules to assist them in resolving specific problems. For example, the mood or energy modules are suggested when experiencing low mood or persistent symptoms of fatigue. The 6 modules of MyDiaMate differ in terms of richness of content and reading time, and thus the participants’ effort required to complete. The estimated reading time for the modules varies between 5 minutes for hypos and 34 minutes for energy. The latter was developed to be followed over several weeks and to be stopped depending on progress in reducing symptoms of fatigue.

### Study Procedure

Between March 2021 and December 2022, MyDiaMate was offered freely to adults with T1D in the Netherlands via the Minddistrict platform. The launch of the app was announced via different diabetes organizations in the Netherlands on their websites and social media platforms. Health care professionals within our network were informed about the possibility of joining the study. We developed a website with information on MyDiaMate for potential users, health care professionals, and the general public. It was used as a hub to link to the Minddistrict platform, where an individual account could be created. Certified health care professionals could request access via email to a separate platform to get acquainted with MyDiaMate outside the study. Information on the duration of the MyDiaMate project, ending in December 2022, was mentioned on the website.

### Ethical Considerations

After signing informed consent, at the start of MyDiaMate, users could volunteer to participate in the user profile study. Participation required filling out a set of questionnaires at the start and filling out an evaluation questionnaire 2 months after creating a MyDiaMate account. The questionnaires were sent out via the secured survey platform Castor Electronic Data Capture (EDC). For technical issues, participants could email the research coordinator (JE). The study protocol was approved by the Medical Ethics Committee of VU University Medical Center (2021.0007).

### Outcome Measures

#### Uptake

Uptake of MyDiaMate was registered by observing the number of monthly created MyDiaMate accounts.

#### Usage

The usage of MyDiaMate was studied based on user log data. A user of MyDiaMate was defined as having an account and having at least opened the starting module diabetes in balance. The participation rate was determined by assessing the number of users who opened the first page of each module, based on the total number of app users for each release. The completion rate was determined by evaluating the number of users who opened the final page of each module, based on the number of users who opened each module.

#### User Profile

For those who consented to take part in the user profile study, we collected sociodemographic data (age, sex, education, and living status), history or current psychological symptoms, and current psychological treatment at baseline. Furthermore, we measured emotional well-being with the 5-item “World Health Organization Well-being Index” (WHO-5) [21]. Diabetes distress was measured with the 11-item “Problem Areas in Diabetes” questionnaire (PAID-11) [22]. Fatigue was measured with the 8-item fatigue severity subscale of the “Checklist Individual Strength” (CIS) questionnaire [23]. A WHO-5 score of less than 50 (range 0-100), indicates poor emotional well-being [21], a PAID-11 score of score of 18 or higher (range 0-44) suggests elevated diabetes distress [22] and a CIS subscale score of 35 or higher (range 8-56) indicates severe fatigue [5].

#### Usage by Profile

Participation and completion rates of diabetes in balance, mood, and energy were determined for all study participants (scoring above and below the cutoffs: PAID-11 score≥18 [diabetes distress], WHO-5 score≤50 [emotional well-being], and CIS score≥35 [fatigue]).

#### User Experiences

To assess experience and satisfaction with MyDiaMate, we measured user expectations at the start and user-friendliness, satisfaction with the number of notifications linked to the mood and energy diary, and clarity of instructions at follow-up. Likert scales ranging from 1 “completely disagree” to 5 “completely agree” were used, with higher scores indicating higher satisfaction. Participants were asked to grade MyDiaMate on a scale from 1 to 10, with higher scores representing higher appreciation. We used an open-ended question to ask for any remarks or recommendations for further improvement.

### Statistical Analysis

The usage of MyDiaMate was summarized using descriptive statistics. Baseline measures of the user profiles were summarized using mean and SD or frequencies and percentages in the case of categorical data. The answers to the open-ended question regarding the user experience were thematically grouped. The chi-square tests were used to look at differences between study participants scoring above and below the PAID-11, WHO-5, and CIS cutoff points in terms of how many of them opened the first and final page of each module. SPSS (version 28.0; IBM Corp) was used to conduct the analyses.

## Results

### Uptake

In total, 1008 people created a MyDiaMate account. Among them, 798 accounts were created during the first release of MyDiaMate, with 497 accounts initiated within the first month. The second release saw 210 new accounts, of which 41 accounts were created in the first month following the release. There was a steady increase of new MyDiaMate accounts each month.

### Usage

Out of the 1008 persons that created an account, 926 actually opened the first module and were classified as users. Their usage data are displayed in Table 1. A total of 726 accounts opened the default module diabetes in balance during the first release and 200 opened diabetes in balance during the second release of MyDiaMate.

**Table 1:** Usage data (participation rate and completion rate; n=926).

Module		Values (number of pages/estimated reading time in minutes)	Opened (participation), n (%)	Closed (completion)^a^, n (%)
**First release (n=726)**				
	Diabetes in balance	37/31	726 (100.0)	207 (28.5)
	Mood	38/24	124 (17.1)	30 (24.2)
	Energy	55/34	142 (19.6)	9 (6.3)
**Second release (n=200)**				
	Diabetes in balance	26/24	200 (100.0)	62 (31.0)
	Social environment	9/6	19 (9.5)	12 (63.2)
	Hypos	10/5	35 (17.5)	24 (68.6)
	Mood	38/24	23 (11.5)	6 (26.0)
	Energy	55/34	23 (11.5)	1 (4.3)
	Food and Feelings	14/11	46 (23.0)	23 (50.0)
	My Goals	1/0.5	167 (18.0)	N/A^b^
	Mood diary	1/0.5	133 (14.4)	N/A
	Energy diary	1/0.5	5 (0.5)	N/A

^a^Based on the number of users who opened the associated module, the completion rate estimates the percentage of users who completed the module.

^b^N/A: not applicable.

### User Profile

Table 2 provides the demographic and diabetes-related characteristics of the participants in the user profile study. Most participants were female (n=217, 63.3%) and higher educated (n=198, 57.6%). The mean age was 43 (SD 14.9; 18-76) years. The majority had been living with T1D for over 5 years (n=241, 73.5%). Of the study participants, 59.1% (n=199) reported low emotional well-being (WHO-5 score≤50), 70.9% (n=239) reported elevated diabetes distress (PAID-11 score≥18), and 52.4% (n=178) reported severe fatigue (CIS score≥35); 21.9% (n=75) reported to currently receiving psychological treatment. Of those participants who reported currently not receiving psychological treatment (78.1%, n=267), 171 (50.7%) participants reported elevated diabetes distress, 141 (41.8%) participants reported low emotional well-being, and 127 (37.4%) participants reported severe fatigue. Over 60% (n=208) stated that the diabetes care team pays enough attention to their feelings with regard to diabetes. As to the expectations regarding MyDiaMate, all precoded responses were endorsed, with the highest for “gaining new insights” and “helping me cope better with diabetes.”

**Table 2:** Demographic and diabetes-related characteristics of participants of the user profile study.

Characteristics		Participants
**Age (n=342; years)**		
	Mean (SD)	43 (14.9)
	Range	18-76
**Sex (n=343), n (%)**		
	Female	217 (63.3)
	Different	1 (0.3)
**Educational level (n=343), n (%)**		
	Lower secondary education	3 (0.9)
	Higher secondary education	129 (37.5)
	Secondary vocational education	13 (3.8)
	Tertiary education (bachelor, master, or equivalent)	198 (57.6)
**Living status (n=343), n (%)**		
	Alone	57 (16.6)
	With 1 or more persons	286 (83.1)
**Time of diagnosis of type 1 diabetes^a^ (n=328), n (%)**		
	Less than 12 months ago	24 (7.3)
	1 to 3 years ago	37 (11.3)
	3 to 5 years ago	26(7.9)
	Longer than 5 years ago	241 (73.5)
**How did you hear about MyDiaMate? (Multiple answers possible), n**		
	Health professional	38
	Social media	162
	MyDiaMate website	118
	Friend, family, or acquaintance	32
**I am worried about my diabetes regulation (n=342), n (%)**		
	I strongly agree	72 (20.9)
	I agree	173 (50.3)
	I disagree	83 (24.1)
	I strongly disagree	14 (4.1)
**I expect MyDiaMate to...(multiple answers possible), n**		
	To help me relax	127
	To help me regain energy	137
	To improve my mood	122
	To help me better cope with diabetes	166
	To gain new insights	170
**I am currently undergoing treatment for psychological complaints (n=342), n (%)**		
	Yes	75 (21.9)
	No	267 (78.1)
**My diabetes care team pays enough attention to my feelings with regard to diabetes (n=342), n** **(%)**		
	Yes	208 (60.8)
	No	134 (39.2)
**Elevated scores of baseline questionnaires, n (%)**		
	WHO-5^b^ (≤50; n=337)	199 (59.1)
	PAID-11^c^ (≥18; n=337)	239 (70.9)
	CIS^d^ (≥35; n=340)	178 (52.4)

^a^14 participants reported having a different type of diabetes.

^b^WHO-5: World Health Organization Well-being Index.

^c^PAID-11: Problem Areas in Diabetes.

^d^CIS: Checklist Individual Strength.

### Usage by User Profile

Due to the small sample size, data from the second release was excluded from the analyses concerning the link between user profiles and usage data. See Multimedia Appendix 1 for the user profile of study participants from the first release and the second release. Tables 3-5 show data from usage of diabetes in balance, mood, and energy, during the first release, differentiating between participants scoring above and below the cutoffs of PAID-11 (diabetes distress), WHO-5 (emotional well-being), and CIS (fatigue). The chi-square tests showed no significant differences for the usage of study participants scoring above and below the cutoffs, in terms of participation and completion rates.

**Table 3:** Usage data (participation rate and completion rate) of participants stratified by diabetes distress (PAID-11^a^ cutoff score ≥18^b^; n=289).

Modules	First page opened (participation), PAID-11 score					Final page opened (completion), PAID-11 score			
	≥18 (n=203), n (%)	<18 (n=86), n (%)	Chi-square (*df*)	*P* value	≥18 (n=203), n (%)		<18 (n=86), n (%)	Chi-square (*df*)	*P* value
Diabetes in balance	203 (100)	86 (100)	N/A^c^	N/A	79 (38.9)		37 (43)	0.4 (1)	.52
Mood	44 (21.7)	15 (17.4)	0.7 (1)	.41	12 (5.9)		5 (5.8)	0.7 (1)	.20
Energy	51 (25.1)	14 (16.3)	2.7 (1)	.10	18 (8.9)		4 (10.8)	0.2 (1)	.64

^a^PAID-11: Problem Areas in Diabetes.

^b^PAID-11 ≥18 indicates elevated diabetes distress.

^c^N/A: not applicable.

**Table 4:** Usage data (participation rate and completion rate) of participants stratified by emotional well-being (WHO-5^a^ cutoff score ≤50^b^; n=289).

Modules	First page opened (participation), WHO-5 score					Final page opened (completion), WHO-5 score			
	≤50 (n=170), n (%)	>50 (n=119), n (%)	Chi-square (*df*)	*P* value	≤50 (n=170), n (%)		>50 (n=119), n (%)	Chi-square (*df*)	*P* value
Diabetes in balance	170 (100)	119 (100)	N/A^c^	N/A	61 (35.8)		55 (46.6)	3.1 (1)	.08
Mood	37 (21.8)	22 (18.6)	0.5 (1)	.50	10 (5.9)		7 (5.9)	0.2 (1)	.69
Energy	26 (15.3)	39 (33.1)	0.0 (1)	.83	15 (8.8)		7 (5.9)	0.9 (1)	.34

^a^WHO-5: World Health Organization Well-being Index.

^b^WHO ≤50 indicates poor emotional well-being.

^c^N/A: not applicable.

**Table 5:** Usage data (participation rate and completion rate) of participants stratified by fatigue (CIS^a^ cutoff score ≥35^b^; n=291).

Modules	First page opened (participation), CIS score					Final page opened (completion), CIS score			
	≥35 (n=147), n (%)	<35 (n=144), n (%)	Chi-square (*df*)	*P* value	≥35 (n=147), n (%)		<35 (n=144), n (%)	Chi-square (*df*)	*P* value
Diabetes in balance	147 (100)	144 (100)	N/A^c^	N/A	55 (37.4)		61 (42.4)	0.7 (1)	.39
Mood	30 (20.4)	29 (20.1)	0.1 (1)	.82	8 (5.4)		9 (6.3)	0.0 (1)	.84
Energy	31 (21.1)	33 (22.9)	0.1 (1)	.71	13 (8.8)		9 (6.3)	1.5 (1)	.22

^a^CIS: Checklist Individual Strength.

^b^CIS ≥35 indicates severe fatigue.

^c^N/A: not applicable.

### User Experiences

Not a single technical problem was reported. A total of 53 study participants made use of the option to provide us with their feedback. MyDiaMate was rated with a median of 6.5 (IQR 6-8; range 3-9) on a 1-10 scale. Suggestions for further development of the app included shortening the amount of text, simplifying the text, and including more clarifying examples and video materials, along with the suggestions to offer reminders within modules and to further explore options for personalization within MyDiaMate.

## Discussion

### Principal Findings

Here we presented the results of a real-world study on the uptake and use of MyDiaMate, which was offered freely to adults with T1D in the Netherlands for a period of 21 months. We collected data on user profiles and user experiences of a self-selected group of participants. Over nearly 2 years, a total of 1008 unique accounts were created, accounting for roughly 1% of the total population of adults with T1D in the Netherlands [24]. But it should be noted that approximately a third of adults with T1D experience elevated diabetes distress and may benefit from some sort of psychosocial support [1-3]. The number of unique MyDiaMate accounts created each month demonstrates that despite only 2 short promotional campaigns that mostly took place via (social) media channels, we were able to reach a sizable audience. These findings suggest good potential for reaching the population of adults with T1D and coping difficulties. Of note in this context is the fact that MyDiaMate was not in any way integrated into the health care system and indeed only a few users reported having heard about MyDiaMate from their health care provider. We can expect a larger reach of MyDiaMate were it to be embedded in routine diabetes care and actively promoted by clinicians.

Usage data, including participation and completion rates of the modules, showed large variations. Participation rates ranged from 9.5% (n=19) for social environment to 100% (n=726) for diabetes in balance. The latter was accessible from the home page, and opened by default. The other modules may have been opened less frequently for a variety of reasons including low perceived need, and the extra effort required to open the modules, as users have to navigate to the catalog and open the module on a different page. Completion rates ranged from 4.3% (n=1) for energy, which is an extensive cognitive behavioral therapy module, to 68.6% (n=24) for the shorter module on hypos. This suggests a higher risk of attrition for longer and more intensive modules, at least without offering reminders or guidance. It is well-known that self-guided e–mental health programs run a higher risk of attrition compared to guided intervention, particularly those requiring more effort, that is, motivation from the user side [25]. Of course, we should acknowledge that not completing a module can be a rational choice of the user, in case sufficient progress has been made and, therefore, low perceived need to continue using the module. Since we did not survey our users on this topic, we cannot be certain as to the causes of incompletion. To further our understanding, qualitative interviews with end users should prove helpful. Here it would also be interesting to gain more insight into how and at what time of the day the app is used, and explore the potential of ecological momentary assessment [26].

The engagement (as observed by participation and completion rates) in this study was lower compared to what was found in the feasibility study of MyDiaMate. This difference could be explained by the fact that this study was set out to be naturalistic, fully relying on self-referral, and without a clear presence of the academic institution conducting the study, or a study coordinator. In traditional research settings (such as in the feasibility study) there is a higher chance of recruiting people who already are more likely to adhere to e–mental health interventions, than people in the general population who install and try available interventions “in the wild” [12,27]. Therefore, we cannot rule out the possibility that a proportion of the accounts created were from people who were just curious to see the application, rather than having a real need and the intention to actually invest in working through the various modules. This may have inflated our results.

Indeed, we also found higher mean completion rates of modules in individuals who volunteered to participate in the profile study (those who partly agreed to participate in traditional research), compared to the total user group. Whereas self-selection is intrinsic to a fully self-guided app, the experienced lack of human contact may at least partly be resolved by adding a conversational user interface, that is, a chatbot. While not preferred by all, and issues around psychological distance and trust exist, studies on chatbots in digital mental health applications show promising results [28-30]. Also, adding optional online peer support groups may be helpful in this respect, and deserves to be further explored.

User profile data collected at baseline showed that the majority of the participants expressed concerns about their diabetes regulation, and reported low emotional well-being and high diabetes distress and fatigue, while the vast majority (78.1%) reported they were not receiving psychological treatment at the time. This indicates an unmet need for psychological support. It is not part of this study but it would be interesting to see whether MyDiaMate impacts self-awareness and stimulates participants to seek professional psychological help when needed [31].

Interestingly, in this study, we did not find evidence to suggest that study participants with a more severe profile, that is, lower emotional well-being, greater diabetes distress, or more fatigue symptoms had lower participation or completion rates of the modules than those with a less severe profile. The level of severity has previously been shown not to moderate the efficacy of (guided) online depression treatment in diabetes and apparently is not critical for developing engagement-enhancing strategies, provided the user is sufficiently motivated [32-34].

Finally, we observed a large variety in user experiences and feedback, ranging from tips on how to shorten the amount of text, to adding more text examples and videos. Additionally, we noted that users’ expectations for MyDiaMate varied, which might explain the variance in the satisfaction ratings of the app. Accommodating individuals’ wishes and needs speaks to the relevance of personalization which is a challenge with a fully self-guided application such as MyDiaMate. Clearly, tailoring content and reminders to users’ individual preferences is key and deserves further research [35]. To this purpose, a baseline assessment of problem areas and preferences could help to offer personalized advice on which modules of MyDiaMate might be most relevant and customized reminders.

### Strengths and Limitations

We succeeded in conducting a real-world study to demonstrate the potential uptake and usage of the intervention in the target population. This is a strength, given that many internet or mobile-based interventions developed for people with chronic medical conditions strand at the pilot-testing phase [36]. Our study has some limitations that are worth mentioning.

First, for pragmatic reasons, we decided to release a second version of MyDiaMate to improve our users’ experience during this naturalistic study. Although in line with the principles of iterative development of digital health applications [37], this did complicate data analysis of the total usage and led us to limit part of the analyses to the first version.

Second, although we provided health care professionals with the opportunity to test MyDiaMate on a separate platform (and 69 professionals made use of this), we cannot rule out the possibility that more health care professionals and others without T1D created a MyDiaMate account and that, therefore, their usage data are included in the analyses.

Third, we only collected data on user profiles of roughly a third of the total group of MyDiaMate users. We should, therefore, be cautious in generalizing our findings to the larger audience, although user behaviors (based on log data) did not appear to be different from the total group. Also, for self-guided interventions, dissemination through web-based marketing appears to be considerably more efficient and cost-effective than dissemination through clinics or pharmacies [38]. MyDiaMate was, therefore, mostly advertised through social media, attracting predominantly those individuals who engage active on such platforms. This was further supported by the survey, indicating that the majority of users were informed about the app through social media, while only a small group learned about it from their health providers. Furthermore, as in many internet-based intervention programs, higher educated people and women were overrepresented in the user-profile study, limiting external validity [38].

To expand and broaden future dissemination, efforts should be made to reach a more diverse user group, taking eHealth literacy, socioeconomic status, and ethnicity into consideration. Health care providers can play a significant role in promoting the use of MyDiaMate supplementing routine diabetes care. For the maintainability of the app, reimbursement should be in place, preferably as an integral part of diabetes care. Alternatively, the app could be made accessible to consumers at a low price to cover maintenance costs and updates. Clearly, demonstrating cost-effectiveness should help to convince health authorities to financially compensate use of the application. eHealth literacy is an important factor to take into consideration when aiming to maximize the reach of a self-guided web-based intervention such as MyDiaMate. This would call for targeted promotional activities to increase the uptake and involvement of a diverse user group in further improving the cultural validity of the intervention [39].

Finally, the study did not set out to evaluate the intervention’s efficacy, which is now the next step, also looking into potential moderators and mediators of effectiveness. Evidence of efficacy will be important to help gain reimbursement and foster the dissemination of MyDiaMate on a larger scale. Here we need to recognize that given the level of distress of the target population, small effects are to be expected, that however, are likely to have clinical relevance from a public mental health perspective [40].

### Conclusions

The findings of this naturalistic study demonstrate the potential of MyDiaMate as a self-help tool for adults with T1D supplementary to ongoing diabetes care, to improve healthy coping with diabetes and mental health. Future research is warranted to explore effective strategies to enhance engagement with the app and test the efficacy of MyDiaMate in a randomized controlled trial.

## Appendix

Multimedia Appendix 1 Demographic and diabetes-related characteristics of participants of the user profile study, releases 1 and 2.

## Abbreviations

CIS: Checklist Individual Strength
EDC: Electronic Data Capture
PAID-11: Problem Areas in Diabetes
T1D: type 1 diabetes
WHO-5: World Health Organization Well-being Index
