# Supplementary material for: A Self-Guided Web-Based App (MyDiaMate) for Enhancing Mental Health in Adults With Type 1 Diabetes: Insights From a Real-World Study in the Netherlands
Source: JMIR Diabetes. 2024 Apr 3;9:e52923. doi: 10.2196/52923 (PMC11024740; doi:10.2196/52923)
Supplement: Multimedia Appendix 1 [file diabetes_v9i1e52923_app1.docx]

**Table S1.** Demographic and diabetes-related characteristics of participants of the user profile study, release 1

| Characteristics | | Participants |
| --- | --- | --- |
| Age in years, mean (SD), (range) | | 43 (14.77), (18-76) |
| Female, n (%), n=294 | | 188 (63.9) |
| **Educational level, n (%), n=294** | |  |
|  | Lower secondary education | 2 (0.7) |
|  | Higher secondary education | 12 (4.1) |
|  | Secondary vocational education | 113 (38) |
|  | Tertiary education (bachelor, master or equivalent) | 167 (56.8) |
| **Living status, n (%), n=294** | |  |
|  | Alone | 48 (16.3) |
|  | Together with one or more persons | 246 (83.7) |
| **Time of diagnosis of type 1 diabetes n (%) *, n=293** | |  |
|  | Less than 12 months ago | 19 (6.7) |
|  | 1 to 3 years ago | 34 (12.1) |
|  | 3 to 5 years ago | 20 (7.1) |
|  | Longer than 5 years ago | 209 (74. 1) |
| **How did you hear about MyDiaMate? (multiple answers possible), n** | |  |
|  | Health professional | 29 |
|  | Social media | 146 |
|  | MyDiaMate website | 95 |
|  | Friend/family/acquaintance | 30 |
| **I am worried about my diabetes regulation, n, n=293** | |  |
|  | I strongly agree | 62 (21.2) |
|  | I agree | 147 (50.2) |
|  | I disagree | 70 (23.9) |
|  | I strongly disagree | 14 (4.8) |
| **I expect MyDiaMate to… (multiple answers possible), n** | |  |
|  | To help me relax | 122 |
|  | To help me regain energy | 137 |
|  | To improve my mood | 248 |
|  | To help me better cope with diabetes | 178 |
|  | To gain new insights | 169 |
| **I am currently undergoing treatment for psychological complaints, n, n=293** | |  |
|  | Yes | 62 (21.2) |
|  | No | 231 (78.8) |
| **My diabetes care team pays enough attention to my feelings with regard to diabetes, n, n=293** | | |
|  | Yes | 175 (59.7) |
|  | No | 118 (40.3) |
| **Elevated scores of baseline questionnaires, n (%)** | |  |
|  | WHO-5 (≤50), n=289 | 170 (58.8) |
|  | PAID-11 (≥18), n=289 | 203 (70.2) |
|  | CIS (≥35), n=291 | 147 (50.5) |

* 11 participants reported having a different type of diabetes

**Table S2.** Demographic and diabetes-related characteristics of participants of the user profile study, release 2

| Characteristics | | Participants |
| --- | --- | --- |
| Age in years, mean (SD), (range), n=51 | | 44.6 (15.72), (18-76) |
| Female, n = 51 (%) | | 31 (59.6) |
|  | Different = 1 |  |
| **Educational level, n (%), n=51** | |  |
|  | Lower secondary education | 1 (2) |
|  | Higher secondary education | 1 (2) |
|  | Secondary vocational education | 18 (35.3) |
|  | Tertiary education (bachelor, master or equivalent) | 31 (60.7) |
| **Living status, n *(%), n=51*** | |  |
|  | Alone | 9 (17.6) |
|  | Together with one or more persons | 42 (82.4) |
| **Time of diagnosis of type 1 diabetes n (%) *, n=51** | |  |
|  | Less than 12 months ago | 5 (10.2) |
|  | 1 to 3 years ago | 3 (6.1) |
|  | 3 to 5 years ago | 6 (12.2) |
|  | Longer than 5 years ago | 35 (71.4) |
| **How did you hear about MyDiaMate? (multiple answers possible), n** | |  |
|  | Health professional | 9 |
|  | Social media | 17 |
|  | MyDiaMate website | 24 |
|  | Friend/family/acquaintance | 5 |
| **I am worried about my diabetes regulation, n (%), n=51** | |  |
|  | I strongly agree | 10 (19.1) |
|  | I agree | 28 (53.8) |
|  | I disagree | 13 (25) |
|  | I strongly disagree | 1 (1.9) |
| **I expect MyDiaMate to… (multiple answers possible), n** | |  |
|  | To help me relax | 29 |
|  | To help me regain energy | 23 |
|  | To improve my mood | 20 |
|  | To help me better cope with diabetes | 28 |
|  | To gain new insights | 30 |
| **I am currently undergoing treatment for psychological complaints, n (%), n=51** | |  |
|  | Yes | 13 (25.5) |
|  | No | 38 (74.5) |
| **My diabetes care team pays enough attention to my feelings with regard to diabetes, n (%), n=51** | | |
|  | Yes | 34 (66.7) |
|  | No | 17 (33.3) |
| **Elevated scores of baseline questionnaires, n (%)** | |  |
|  | WHO 5 (≤50) n=50 | 30 (57.7) |
|  | PAID-11 (≥18) n=50 | 38 (73.1) |
|  | CIS (≥35) n=51 | 31 (59.6) |

* 3 participants reported having a different type of diabetes
